# Major obstetric haemorrhage in Metro East, Cape Town, South Africa: a population-based cohort study using the maternal near-miss approach

**DOI:** 10.1186/s12884-019-2668-x

**Published:** 2020-01-06

**Authors:** Anke Heitkamp, Simcha Lot Aronson, Thomas van den Akker, Linda Vollmer, Stefan Gebhardt, Jos van Roosmalen, Johanna I. de Vries, Gerhard Theron

**Affiliations:** 1 Department of Obstetrics and Gynaecology, Stellenbosch University and Tygerberg Academic Hospital, Francie van Zijl Avenue, Cape Town, 7505 South Africa; 2 Department of Obstetrics and Gynaecology, Amsterdam UMC, Vrije Universiteit Amsterdam, Boelelaan 1117, 1081 HV Amsterdam, the Netherlands; 3 Department of Obstetrics and Gynaecology, Leiden University Medical Centre, Albinusdreef 2, 2333 ZA Leiden, the Netherlands; 4 Athena Institute, Vrije Universiteit Amsterdam, Boelelaan 1085, 1081 HV Amsterdam, the Netherlands

**Keywords:** Maternal near-miss, Major obstetric haemorrhage, Placental abruption, Caesarean section

## Abstract

**Background:**

Major obstetric haemorrhage is a leading cause of maternal mortality and accounts for one-third of maternal deaths in of Africa. This study aimed to assess the population-based incidence, causes, management and outcomes of major obstetric haemorrhage and risk factors associated with poor maternal outcome.

**Methods:**

Women with major obstetric haemorrhage who met the WHO maternal near-miss criteria or died in the Metro East region, Cape Town, South Africa, were evaluated from November 2014–November 2015. Major obstetric haemorrhage was defined as haemorrhage in pregnancies of at least 20 weeks’ gestation or occurring up to 42 days after birth, and leading to hysterectomy, hypovolaemic shock or blood transfusion of ≥5 units of Packed Red Blood Cells. A logistic regression model was used to analyse associations with poor outcome, defined as major obstetric haemorrhage leading to massive transfusion of ≥8 units of packed red blood cells, hysterectomy or death.

**Results:**

The incidence of major obstetric haemorrhage was 3/1000 births, and the incidence of massive transfusion was 4/10.000 births in the Metro East region (32.862 births occurred during the studied time period). Leading causes of haemorrhage were placental abruption 45/119 (37.8%), complications of caesarean section 29/119 (24.4%) and uterine atony 13/119 (10.9%). Therapeutic oxytocin was administered in 98/119 (82.4%) women and hysterectomy performed in 33/119 (27.7%). The median numbers of packed red blood cells and units of Fresh Frozen Plasma transfused were 6 (interquartile range 4–7) and 3 (interquartile range 2–4), ratio 1.7:1. Caesarean section was independently associated with poor maternal outcome: adjusted OR 4.01 [95% CI 1.58, 10.14].

**Conclusions:**

Assessment of major obstetric haemorrhage using the Maternal Near Miss approach revealed that placental abruption and complications of caesarean section were the major causes of major obstetric haemorrhage. Caesarean section was associated with poor outcome.

## Background

Major obstetric haemorrhage (MOH), mostly occurring postpartum, is a leading cause of maternal mortality worldwide and accounts for one-third of maternal deaths in Africa [1–3]. MOH is associated with severe maternal morbidity, including severe anaemia, disseminated intravascular coagulation, shock, multi-organ failure and hysterectomy, as well as long-term psychological trauma [4–7]. The disparity of maternal deaths (MD) between low/middle-income and high-income countries is evident and reflects, among others, differences in the quality of obstetric care [2, 5, 8, 9]. Multiple factors contribute to these differences, including timely access to emergency interventions, availability of trained healthcare staff, financial and infrastructural factors [5, 9, 10].

According to the ‘National Committee on Confidential Enquiries into Maternal Deaths’, there was a 40% increase in deaths due to MOH from 5 to 7 per 100.000 live births in the Western Cape province from 2011 to 2013 and 2014–2016, corresponding with two triennia reported by the ‘National Committee on Confidential Enquiries into Maternal Deaths’. This increase in MOH in the Western Cape contrasts with the decreasing trend in MOH-related deaths in the whole of South Africa. It was considered an important worrying epidemiological finding meriting closer investigation [11, 12].

Given the Sustainable Development Goals’ aim to reduce maternal mortality, it is essential to focus on effective prevention, early diagnosis and improved clinical management of MOH in this region [13].

The World Health Organization (WHO) maternal near-miss (MNM) approach (2011) was developed to assess and improve obstetric care by identifying women who nearly died, but survived severe complications of pregnancy [14]. Measuring MNM in addition to MD, facilitates the investigation of a considerably larger group of women, identification of factors associated with MOH and evaluation of health care interventions [14, 15].

This study examined women with MOH who met the WHO organ dysfunction-based MNM criteria or died. Aims were to assess the incidence, case fatality rate, causes, management and outcomes of MOH as well as to examine factors associated with poor outcome, and evaluate clinical practice regarding massive blood transfusion in the Metro East region, South Africa. In the near future, this may contribute to revision of protocols in the attempt to further reduce severe maternal outcome by MOH.

## Methods

A population-based cohort study was designed to describe organ dysfunction-based MNM and MD due to major obstetric haemorrhage (MOH) in the public health sector in the Metro East region, Cape Town, Western Cape province, South Africa. From 1 November 2014 to 1 November 2015, women admitted to Tygerberg Hospital (TBH) were selected who met the MNM criteria or died and were suffering from MOH after a gestational age of 20 weeks. In South Africa and in accordance with the ‘National Committee on Confidential Enquiries into Maternal Deaths’, antepartum haemorrhage is defined as bleeding after 20 weeks gestation. Bleeding prior to this gestation is classified as miscarriage and early pregnancy loss and not as MOH.

Women who met the MNM criteria at TBH, were included. TBH is a public tertiary care facility in Cape Town as well as serving as the academic hospital of the Faculty of Medicine and Health Sciences of Stellenbosch University, and the referral hospital for Metro East region. TBH has a well-equipped blood bank as well surgical and medical intensive care units. Women with pregnancy related critical care problems are treated in the Obstetric Critical Care Unit in the Department of Obstetrics.

The referral system in Metro East region is organized according to a three-tiered referral system with midwife-led units being level 0 for women with low-risk pregnancies. Depending degree of pregnancy related risk, women will be referred to district hospitals being level 1 or TBH which serves as both the second and third line referral centre.

To investigate whether there might have been missing inclusions of MNM at level 1 hospitals, a three-months survey was conducted at these institutions, revealing only three cases. These three women were not included in this study, because of difficulties in tracing the files.

Besides women who are referred from the Metro East region, TBH also receives some of the most complicated cases from other regions within the wider Western Cape province. In order to calculate accurate incidence rates, we only used data from patients referred within the Metro East region and not from the entire Western Cape Province.

MNM was defined as a woman who nearly died but survived a complication that occurred during pregnancy, childbirth or within 42 days after birth or termination of pregnancy [14]. MOH was defined as haemorrhage, occurring antepartum, intrapartum or postpartum, and leading to hysterectomy or blood transfusion ≥5 units of Packed Red Blood Cells (RBCs) or hypovolemic shock, all of which are considered as markers of MNM [15]. Estimated volume of blood loss was not included in the definition to avoid the inaccuracies associated with visual estimation [16]. Poor maternal outcome was defined as MOH followed by massive blood transfusion (defined as ≥8 units of RBCs) or hysterectomy or MD [17, 18].

Women with MNM were identified on a daily basis in the obstetric department by accessing admission and referral books by one of the investigators (AH). All healthcare workers (consultants, registrars, medical officers, nurses, interns, students) were requested to inform the investigator when a woman who met the MNM criteria was admitted. Medical files of these women were screened by two investigators (LV, AH) using the WHO organ dysfunction-based MNM criteria [14]. Women who met the definition of MOH as above were included in the data collection. Data collection was performed using a form specifically designed for this study including demographic details, and events before, during and after birth, up to 42 days postpartum. All the patient data were de-identified upon data collection and were collected from patient records at TBH and from the files stored electronically on the protected TBH OpenText Enterprise Content Management system. Incomplete data were attempted to be completed by searching in the stored original paper files.

TBH has protocols for postpartum haemorrhage and severe haemorrhage based on international guidelines, including recommendations on how to administer blood products. Interventions such as uterine massage and urinary catheterization were not assessed because, although these interventions are considered standard procedures, most were not recorded. Arterial embolization and cell saving equipment were not available.

### Statistical analyses

Data were analysed using SPSS version 23.0 (IBM Corp. Released 2015). Descriptive data are presented as frequencies, percentages, means with standard deviations if normally distributed and medians with interquartile ranges (IQR) for skewed data. Associations of poor outcome with maternal and obstetric risk indicators were analysed in a multivariable logistic regression model using risk factors for MOH from the literature [19]. Cross-tabulations were used to determine whether there was a significant relationship between the explanatory variable and poor outcome. In the multivariable logistic regression model, adjusted odd ratios (OR) for risk factors related to birth were determined, corrected for antenatal risk factors. A maximum of four antenatal risk factors were included to ensure that the dataset contained at least ten women for each variable. In the multivariable logistic regression, *P*-values < 0.05 were considered statistically significant.

The four antenatal factors were checked for collinearity. Collinearity was determined to be low if the correlation was between 0 and 0.3.

## Results

The number of women who gave birth in the Metro East region during the one-year study period was 32.862. We identified 119 women who experienced MOH, of which one woman died. In these 119 women, 112 singletons and 7 twins were born. The incidence of MNM with MOH was 3 per 1000 births, and the incidence of massive blood transfusion due to MOH was 4 per 10.000 births.

The majority of cases (95/119 (75.6%)) were referred to TBH from a midwife-led unit or level 1 hospital. The mean age of included women was 28 ± 6.5 SD (16–43) years, and the median BMI was 26 kg/m^2^ (range 23–31). The median gestational age was 36 weeks (range 32–39), with 61/119 (51.2%) women having preterm birth (Table 1).

**Table 1:** Characteristics of women with MOH (*n* = 119)

	N (%)
Age at delivery (years)	
< 20	11 (9.2)
20–34	88 (73.9)
35–39	10 (8.4)
≥ 40	10 (8.4)
BMI	
< 18	2 (1.7)
18–24	36 (30.3)
25–29	31 (26.1)
30–34	12 (10.1)
≥ 35	20 (16.8)
Missing data	18 (15.1)
Parity^a^	
0	37 (31.1)
1–3	76 (63.9)
≥ 4	6 (5.0)
Gestational age at delivery (weeks)	
20–24	2 (1.7)
24–31	26 (21.8)
32–37	33 (27.7)
Full term (> 37)	52 (43.7)
Missing data	6 (5.0)

^a^Number of previous births

Of all women with MOH, 46/119 (38.7%) had poor maternal outcome, of whom 25/46 (54.4%) underwent hysterectomy, 12 received massive blood transfusions, eight had both and one woman died (Table 2).

**Table 2:** Obstetric outcomes of women with MOH (*n* = 119)

	N (%)
Mode of birth	
Spontaneous vaginal birth	56 (47.1)
Instrumental	4 (3.3)
Caesarean section	58 (48.7)
Laparotomy for ectopic pregnancy	1 (0.8)
Maternal outcome	
Maternal death	1 (0.8)
Poor outcome MOH^a^	46 (38.7)
Case fatality rate	1 (0.8)
Neonatal outcome (*n* = 126)	
Live births	61 (48.8)
Stillbirths	58 (46.0)
Neonatal deaths	3 (2.4)
Missing data	4 (3.2)

^a^poor outcome = massive blood transfusion of ≥8 units of RBCs, hysterectomy or death

MOH occurred antepartum in 12/119 (10.1%), intrapartum in 47/119 (39.5%) and postpartum in 60/119 women (50.4%).

The leading cause of MOH was placental abruption in 45 women (37.8%), followed by complications related to caesarean section (CS) (vascular and tissue related, not placenta related) in 29 (24.4%) (Table 3). Characteristics and outcomes of women with placental abruption are shown in Table 4. The mean age (SD) was 25.7 ± 5.9 SD years and the median gestational age was 33 (29–36) weeks. No cocaine use or multiple pregnancies were reported in these 45 women.

**Table 3:** Primary causes of obstetric haemorrhage (*n* = 119)

	N (%)
Placental abnormalities	
Abruption	
with hypertension	35 (29.4)
without hypertension	10 (8.4)
Retained placenta	11 (9.2)
Placenta previa	5 (4.2)
Abnormally invasive placenta	5 (4.2)
Operative complications	
during CS^a^ after CS	10 (8.4) 19 (16)
Uterine Atony	13 (10.9)
Perineal trauma	6
Uterine rupture	3 (2.5)
Uterine inversion Subcapsular liver haematoma	1 (0.8) 1 (0.8)

^a^*CS* caesarean section

**Table 4:** Characteristics and outcomes of women with placental abruption as primary cause of MOH [20] (*n* = 45)

	N (%)
*Maternal risk factors*	
Primigravida	15 (33.3)
Parity ≥4	2 (4.4)
Alcohol^a^	7 (2)
Smoking^a^	11 (25)
Anaemia	2 (4.4)
*Risk factors from past obstetrics history*	
Hypertensive disease	12 (26.7)
Caesarean section	8 (17.8)
Placental abruption	3 (6.7)
Stillbirth	4 (8.9)
Neonatal death	3 (6.7)
*Pregnancy-associated risk factors*	
Hypertension	
(Pregnancy induced) hypertension	7 (15.6)
Pre-eclampsia	28 (62.2)
Gestation	
20–24	5 (11.1)
25–31	11 (24.4)
32–37	19 (42.2)
> 37	7 (15.6)
Missing	3 (6.7)
*Outcomes*	
Stillbirths	42 (95)
Stillbirths on admission	35 (77.8)
Fresh stillbirths	7 (15.6)
CS^b^ for suspected abruption (baby alive before CS)	5 (11.1)
CS with stillbirth	7 (15.6)
Hysterectomy	1 (2.2)
Uterine rupture^c^	1 (2.2)
Massive transfusion^d^	5 (11.1)

^*a*^during current pregnancy

^b^*CS* Caesarean section

^c^failed induction for stillbirth, presented with acute abdominal pain during laparotomy uterine rupture was diagnosed, hysterectomy was needed

^d^≥ 8 RBC transfusion

The majority of women (26/45 (57.8%)) presented intrapartum, mostly presenting with disseminated intravascular coagulation or shock, and gave birth some hours later). Ten women (22.2%) presented antepartum and 9 (20%) women presented postpartum.

Interventions in the management of MOH are presented in Table 5. For every woman who had MNM due to MOH, interventions were identified which were performed to control MOH according to local protocol. The chronological order of interventions was not retrievable. Therapeutic oxytocin was administered (prophylactic dose immediate postpartum is routine) most frequently to 98/119 women (82.4%) and was the only intervention in one-quarter of all women. In addition, 34/119 (28.6%) women received more than one uterotonic drug, and 64/119 (53.8%) women received a mechanical or surgical intervention.

**Table 5:** Summary of interventions in the management of MOH by cause^a^

	Atony	Retained	AIP^b^	Abruption	CS compli-cations^c^	Total	
	*N* = 13	*N* = 11	*N* = 10	*N* = 45	*N* = 29	*N* = 107	%
*Medical interventions*							
Oxytocin^d^	13	10	7	35	24	98	82.4
Ergometrine	7	1	3	1	9	23	19.3
Misoprostol	5	3	–	3	7	20	16.8
Tranexamic acid	2	1	2	4	8	18	15.1
Prostaglandin F2-alpha	3	–	1	1	3	8	6.7
*Mechanical intervention*							
Balloon or condom tamponade	6	–	–	1	1	10	8.4
*Surgical interventions*							
Relook laparotomy	5	–	2	2	24	34	28.6
Hysterectomy	6	2	7	1	14	33	27.7
Removal of retained products	3	10	2	4	3	23	19.3
Tear repair in theatre	–	2	–	6	2	16	13.4
B-Lynch suture	5	–	1	3	4	13	10.9
Abdominal packing	2	–	–	1	1	4	3.4
Artery ligation	–	–	–	1	2	3	2.5
Uterine rupture repair	–	–	–	–	–	1	0.8
Uterine inversion reversal	–	–	–	–	–	1	0.8
*Management*							
Transfusion of blood products	13	10	10	45	29	117	98.3
Massive transfusion	1	1	2	5	9	20	16.8
Admission to ICU	2	–	1	1	6	11	9.2
Missing data						3	2.5

^a^the following causes are not displayed in this table: laceration, uterine rupture and uterine inversion

Interventions for these women are included in the total numbers

^b^abnormally invasive placenta, including placenta previa

^c^bleeding related to caesarean section

^d^therapeutic dose (> 10 IE oxytocin) [21]

The ratio of total units of RBCs to total units of Fresh Frozen Plasma was 1.7:1 (686 units/394 units), the median number of RBC per woman was 6 (range 4–6). The ratio in the massive transfusion group was 1.8:1 (207 units/116 units), with a maximum of 41 blood products in one woman.

There were 109 women included in the multivariable logistic regression, because missing data in one of the factors meant some women had to be excluded. CS was independently associated with poor maternal outcome (OR 4.01; 95% CI 1.58, 10.14) (Table 6). Checking for collinearity was done and low correlations between oxytocin and CS (correlation coefficient: − 0,204), induction of labour and CS (correlation coefficient − 0.136) and prolonged labour and CS (correlation coefficient 0.249) were found.

**Table 6:** Univariate analysis and multivariable logistic regression of risk factors associated poor outcome (defined as massive blood transfusion, hysterectomy or death)

Variable	Poor outcome *N* = 46	No poor outcome *N* = 73	Missing data	Crude OR [95% CI]	Adjusted OR [95% CI]
	N (%)	N (%)	N (%)		
*Antenatal*					
BMI ≥ 35	11 (23.9)	9 (12.3)	15 (12.6)	2.09 [0.78, 5.60]	–
Age at delivery ≥35	11 (23.9)	8 (11.0)	0	2.55 [0.94, 6.94]	–
Previous c. section	19 (41.3)	18 (24.7)	3 (2.5)	2.15 [0.97, 4.78]	–
Primigravida	12 (26.1)	21 (46.7)	0	0.87 [0.38, 2.01]	–
*Relating to birth*					
Prolonged labour	9 (19.6)	4 (5.5)	8 (6.7)	4.43 [1.27, 15.47]	3.72 [0.96, 14.37]
Induction of labour	11 (23.9)	30 (41.1)	6 (5.0)	0.56 [0.20, 1.05]	0.46 [0.18, 1.15]
Oxytocin foraugmentation	5 (10.9)	21 (46.7)	9 (7.6)	0.33 [0.12, 0.97]	0.32 [0.10, 1.05]
Caesarean section^a^	34 (73.9)	24 (32.9)	0	5.79 [2.55, 13.13]***	4.01 [1.58, 10.14] **

^a^includes caesarean section on stillbirth

** < *p* 0.01

*** < *p* 0.001

## Discussion

The main cause of MOH in our study was placental abruption. The majority of these women suffered from hypertensive disease of pregnancy and lost their child. Other frequent causes of MOH were complications during or after CS and uterine atony. It is remarkable that only CS was associated with poor maternal outcome. Moreover, uterine rupture was seldom encountered in the overall MNM group.

The reported incidence of MOH is comparable to prior published incidence rates in Pretoria, South Africa in 1998 (3 per 1000 births) and 2001 (2.8 per 1000 births) [22, 23]. Incidence rates in high-income countries are reported to be 2.3 per 1000 births (Ireland in 2011) [24]. The WHO Multicountry Survey on Maternal and New-born Health reported incidence rates for postpartum haemorrhage of 2.9, 1.4 and 2.0 per 1000 births for low, medium and high Human Development Index countries in 2012 [5]. Yet, this survey audited severe maternal outcome (MNM and MD) due to postpartum haemorrhage only, whereas our study included antepartum and intrapartum haemorrhage as well. The incidence of postpartum haemorrhage in our study was 1.9 per 1000 births, comparable to the medium to high Human Development Index countries in the WHO survey. In general, the comparison of incidence rates of obstetric haemorrhage remains difficult, because definitions vary widely in the literature. On the other hand, the case fatality rate of MOH was low (0.9%) and is consistent with the literature [5, 9].

The finding of placental abruption as the main cause of MOH in our population cannot easily be compared with other populations since most studies report antepartum haemorrhage and postpartum haemorrhage separately. The fact that uterine atony is not the most frequent cause for MOH is probably also because our study combines MOH before, during and after birth [9, 25]. This underlines the importance of including ante- and intrapartum bleeding in studies of MOH, rather than limiting their scope to include postpartum haemorrhage only.

Hypertensive disorders of pregnancy, placental abruption and preterm labour are pathophysiologically related with early ischemic placental disease [23, 26].

The fact that stillbirths occurred in 95% of women with abruption was not unexpected, given that the large majority presented already in shock or with symptoms of disseminated intravascular coagulation. Despite knowledge that appropriate management is to deliver stillbirths vaginally, in daily practice this is not always possible, due to acute maternal compromise requiring surgical intervention. In this study, we reported a relatively high number of CS in cases of stillbirth. Indications were maternal condition too poor for induction of labour (3/7), ≥2 previous CS, (1/7, relative contra-indication to induce), failed induction (2/7) or ruptured uterus (1/7).

The high number of complications during and after CS can be related to the risk factors of the women in this population. The high percentage of women with obesity (26.9% BMI ≥30) and hypertensive disorders may complicate CS. Secondly, lack of skilled doctors can be associated with poor maternal outcome after CS [11, 27, 28]. The majority of women with MNM had low-risk pregnancies and were referred from level 1 hospitals with a high patient burden, mainly managed by medical officers and only one or two obstetricians present. Further audit may give more insight into the quality of care in this setting.

With regard to interventions in the management of MOH, low rates of intrauterine balloon tamponade, and administration of tranexamic acid are notable. This can be explained by the fact that, in 2014, tranexamic acid was not yet included in the local guidelines. The Bakri balloon for intrauterine tamponade is a relatively expensive device and therefore rarely utilised at TBH. An alternative and more affordable device, the Ellavi balloon, was developed in South Africa, but only introduced in 2016 [29].

Even though our study suggests that the local protocol for management of MOH was followed, detailed information - e.g., timing, availability, and quality of the interventions and training of staff – will have to be studied in more depth in order to identify opportunities to improve management of MOH.

Because of the increasing trend of MOH in the Western Cape province, recommendations from the National Committee on Confidential Enquiries into Maternal Deaths to implement guidelines that prevent MOH, develop protocols for management of postpartum haemorrhage with tranexamic acid and balloon tamponade and practicing emergency drills, should be prioritised [11]. .Antenatal care and appropriate blood pressure control for hypertensive disease are, however, just as important to prevent MOH and abruptions.

There is no worldwide consensus on blood transfusion protocols, due to a lack of strong evidence, which is a result of discrepancy in definitions, resources and only few available randomized controlled trials [19, 30]. At TBH, the local severe haemorrhage protocol recommends that, if bleeding is not controlled after the administration of 4 units of RBCs, to infuse blood products in a ratio of 1 RBC: 1 Fresh Frozen Plasma: 1 platelets. However, our data does not reflect this recommendation meaning more individualized care was performed in practice.

We importantly adhered strictly to the criterion of ≥5 RBCs for inclusion as MNM. Nonetheless, we also separately assessed massive blood transfusion since this assessment enabled comparisons with studies done in other countries. The incidence of massive transfusion was 40 per 100.000 births and is within the range of the incidence reported for the United Kingdom between 2012 and 2013 (23 per 100.000 births) and the Netherlands between 2011 and 2012 (65 per 100.000 births) [17, 18], using the same definition for massive blood transfusion.

The strength of this study is that nearly all women with MOH from the Metro East region were included in our database as a result of a well-organized referral system. Under reportage was investigated and, in 3 months, only three women with MNM were missed in the level 1 hospitals, meaning 12 were missed in 1 year so that only 3% of cases of MNM were underreported,. Hence, these data are considered population-based at district level. This emphasizes the need and value of a national maternal health care and perinatal data registry, which is not presently available in South Africa. Limitations of the study are that, despite our efforts of daily assessment, either incomplete documentation or inadequate data extraction from handwritten medical files could have led to information bias or non-differential misclassification. For instance, the history of hypertensive disease in pregnancy or previous pregnancies was difficult to trace and the already high prevalence of hypertensive disease of pregnancy during placental abruption can thus still be an underestimation. Although the cause of haemorrhage is likely to be multifactorial, it often involves uterine atony. Since we only documented the primary cause of haemorrhage, there might be an underestimation of the incidence of uterine atony as well.

## Conclusions

Our assessment of MOH-related severe maternal outcome (MNM and MD) shows high rates of morbidity and low rates of mortality, consistent with the literature. The incidence of severe maternal outcome due to MOH is comparable to middle and high-income countries. The leading cause of MOH was placental abruption suggesting a shift towards ante- and intrapartum haemorrhage. This, in the WHO terminology (ICD-MM), illustrates the importance of tracing back the chain of events to antepartum, rather than focusing on postpartum haemorrhage only, which is what often happens in practice. Hypertensive disorders of pregnancy were present in a majority of women with placental abruptions, underlining the association between ischemic placental disease, hypertensive disorders and haemorrhage. Since caesarean section was independently associated with poor maternal outcome, careful consideration and vigilance are needed when deciding on this mode of birth. A focus on developing an evidence-based massive transfusion protocol is important to improve patient safety. Progress remains to be made regarding the reduction of morbidity rates; hence obstetric audit should be encouraged to identify opportunities to improve MOH management in order to avoid hysterectomy, massive transfusion and placental abruption in women with preeclampsia.

## Data Availability

The dataset used and/or analysed during the current study are available from the corresponding author on reasonable request.

## Abbreviations

BMI: Body Mass Index
CS: Caesarean Section
IQR: Inter Quartile Range
MD: Maternal Deaths
MNM: Maternal Near-Miss
MOH: Major Obstetric Haemorrhage
RBC: Red Blood Cells (RBCs
SD: Standard Deviation
TBH: Tygerberg Hospital
WHO: World Health Organization
